# Ulnar lengthening in multiple hereditary exostosis with forearm deformity: a long-term follow-up study

**DOI:** 10.3389/fped.2025.1681144

**Published:** 2025-11-11

**Authors:** Bing Zeng, Han Xiao, An Yan, Kun Liu, Mi Zhou, Haibo Mei

**Affiliations:** 1 Department of Orthopedics, The First Affiliated Hospital of Jinan University, Guangzhou, Guangdong, China; 2 Department of Orthopedics, Changde Hospital, Xiangya School of Medicine, Central South University (the First People’s Hospital of Changde City), Changde, Hunan, China; 3 The Affiliated Children’s Hospital of Xiangya School of Medicine, Central South University (Hunan Children’s Hospital), Changsha, Hunan, China; 4 Hunan Provincial Key Laboratory of Pediatric Orthopedics, Changsha, Hunan, China; 5 Hunan Provincial University Key Laboratory of the Fundamental and Clinical Research on Neurodegenerative Diseases, Changsha Medical University, Changsha, Hunan, China

**Keywords:** multiple hereditary exostosis, ulnar lengthening surgery, radial head dislocation, forearm deformity, Masada classification

## Abstract

**Purpose:**

Ulnar lengthening surgery for multiple hereditary exostosis (MHE) with radial head dislocation can achieve satisfactory reduction in the short term. However, its long-term efficacy remains controversial. This study aims to investigate the long-term effects of ulnar lengthening on forearm deformity, functional improvement, and radial head dislocation in pediatric patients.

**Methods:**

We conducted a retrospective study of patients with MHE who underwent ulnar lengthening procedures at Hunan Children's Hospital between 2010 and 2020. The radial articular angle (RAA), ulnar variance (UV), radial variance (RV), and range of motion of the affected forearm and elbow were clinically assessed before surgery and at the last follow-up. The total ulna lengthening distance (LD) and radiographic outcome were also recorded.

**Results:**

The study included 12 pediatric patients, 6 girls and 6 boys, with a total of 12 forearms affected. The average age was 7.6 ± 2.9 years. The average follow-up time was 63.8 ± 15 months. The mean LD was 2.5 ± 0.7 cm, while the duration of distraction was 141 ± 62.5 days. At the last follow-up, the ulnar length percent, UV, RV, and radial bow (RB) showed statistically significant improvements. The pre-ulnar length percent was 0.91 ± 0.05, while the post-ulnar length percent was 1.0 ± 0.08 (*p* < 0.001). The pre-UV was −1.1 ± 0.5, while the post-UV was −0.4 ± 0.5 (*p* < 0.001). The pre-RB was 8.8 ± 1.2, while the post-RB was 7.6 ± 1.4 (*p* < 0.001). The pre-RV was 0.7 ± 0.6, while the post-RV was 0.4 ± 0.8 (*p* = 0.02). Postoperative assessment revealed improved forearm supination and elbow pronation mobility to varying extents after the ulnar lengthening procedure. The pre-supination was 56° ± 10.0°, while the post-supination was 67° ± 9.4° (*p* < 0.001). The pre-pronation was 54° ± 9.3°, while the post-pronation was 65° ± 7.8° (*p* = 0.003). One pin-track infection was recorded.

**Conclusions:**

Early ulnar lengthening surgery remains a reliable treatment option for patients with MHE who develop severe forearm shortening secondary to ulnar involvement.

## Introduction

Multiple hereditary exostosis (MHE) is a rare autosomal dominant disorder with an estimated prevalence of 1:50,000 in the general population (1). This condition is characterized by the development of multiple osteochondromas, which predominantly occur in the metaphyseal regions of long bones (2). Symptom severity primarily depends on the size and anatomical location of these exostoses, resulting in a wide clinical spectrum of manifestations (3). While some cases remain completely asymptomatic, others present with debilitating symptoms including chronic pain, structural deformities, and functional limitations that can significantly impair daily activities and psychosocial well-being (4).

The forearm is particularly susceptible to growth disturbances due to multiple osteochondromas frequently involving the distal radius and ulna, leading to significant forearm deformity. This pathological process typically manifests through a triad of ulnar shortening, radial bowing, and wrist ulnar deviation, with concomitant radial head dislocation occurring in severe cases (5). Jo et al. (6) found that proportional ulnar shortening <0.9 and radial bowing of 8.1% or greater can be used to predict the risk of radial head dislocation.

Radial head dislocation may lead to marked functional impairment, pain, and deformity (7). Despite advances in surgical techniques, addressing radial head dislocation in children with MHE remains a complex issue for orthopedic surgeons. Ulnar lengthening is currently regarded as an effective surgical approach for managing forearm deformity in MHE patients, particularly in pediatric cases classified as Masada types I and IIb (8). Huang et al. (9) reported 28 cases of successful radial head reduction in a series of 30 forearms treated with ulnar lengthening and concomitant deformity correction. While ulnar lengthening surgery for MHE with radial head dislocation can achieve satisfactory reduction in the short term, it still cannot restore the normal anatomical structure of the humeroradial joint. There is still a lack of literature reports on long-term follow-up. Its long-term efficacy remains controversial.

This study retrospectively analyzed patients with MHE who underwent ulnar lengthening procedures and evaluated their clinical and radiographic outcomes. We aim to investigate the long-term effects of ulnar lengthening on forearm deformity, functional improvement, and radial head dislocation in pediatric patients.

## Patients and methods

### Patients

We conducted a retrospective study of patients with MHE who underwent ulnar lengthening procedures at Hunan Children's Hospital between 2010 and 2020. The inclusion criterion was as follows: a follow-up period of >5 years after ulnar lengthening surgery or to skeletal maturity and patients undergoing an immediate single-bone forearm procedure after ulnar lengthening. Ultimately, 12 patients were enrolled in the study. Forearm deformities were morphologically assessed according to Jo et al.’s classification (6). The indication for ulnar lengthening surgery was that significant ulnar shortening (ulnar length percent <1.0) accompanied by marked forearm deformity and functional impairment. The quality of the radiocapitellar joint status was classified as good, fair, or poor according to a previous report by a senior physician (Table 1) (9). The study was approved by the Ethics Committee of the Hunan Children's Hospital. All patient parents and/or legal guardians involved in this study gave informed written consent to participate.

**Table 1:** Quality of radial head reduction.

Quality	Description
Good	The long axis of the proximal radial shaft passes through the middle 1/3 of the capitellum on anteroposterior and lateral views
Fair	The long axis of the proximal radial shaft passes through the capitellum but not through its middle 1/3
Poor	The long axis of the proximal radial shaft does not pass through the capitellum on anteroposterior or lateral views

### Operative technique

According to the site of ulnar deformity, we selected the proximal one-third to mid-shaft of the ulna as the osteotomy plane. Under C-arm fluoroscopy, the ulnar osteotomy position was identified. A longitudinal dorsal incision approximately 4 cm in length was made to expose the ulnar subperiosteum and perform the osteotomy. A 2.0 mm Kirschner wire was inserted into the ulna to stabilize the proximal and distal segments of the osteotomy. The Ilizarov frame was assembled, with crossed Kirschner wires inserted proximally and distally in the ulna and connected to the frame for fixation, while ulnar deformity correction was performed simultaneously. Fluoroscopic confirmation ensured adequate fixation and correction of the ulnar angular deformity. The exostosis at the distal ulna or radius was excised concurrently or during the follow-up, with particular attention paid to preserving the distal growth cartilage, as it was causing restricted pronation–supination and discomfort. The surface of the resection was protected by bone wax. Ulnar distraction was initiated 7 days postoperatively at a rate of 1 mm/day, administered in four divided sessions of lengthening each day.

### Radial and clinical assessment

**Figure 1:**
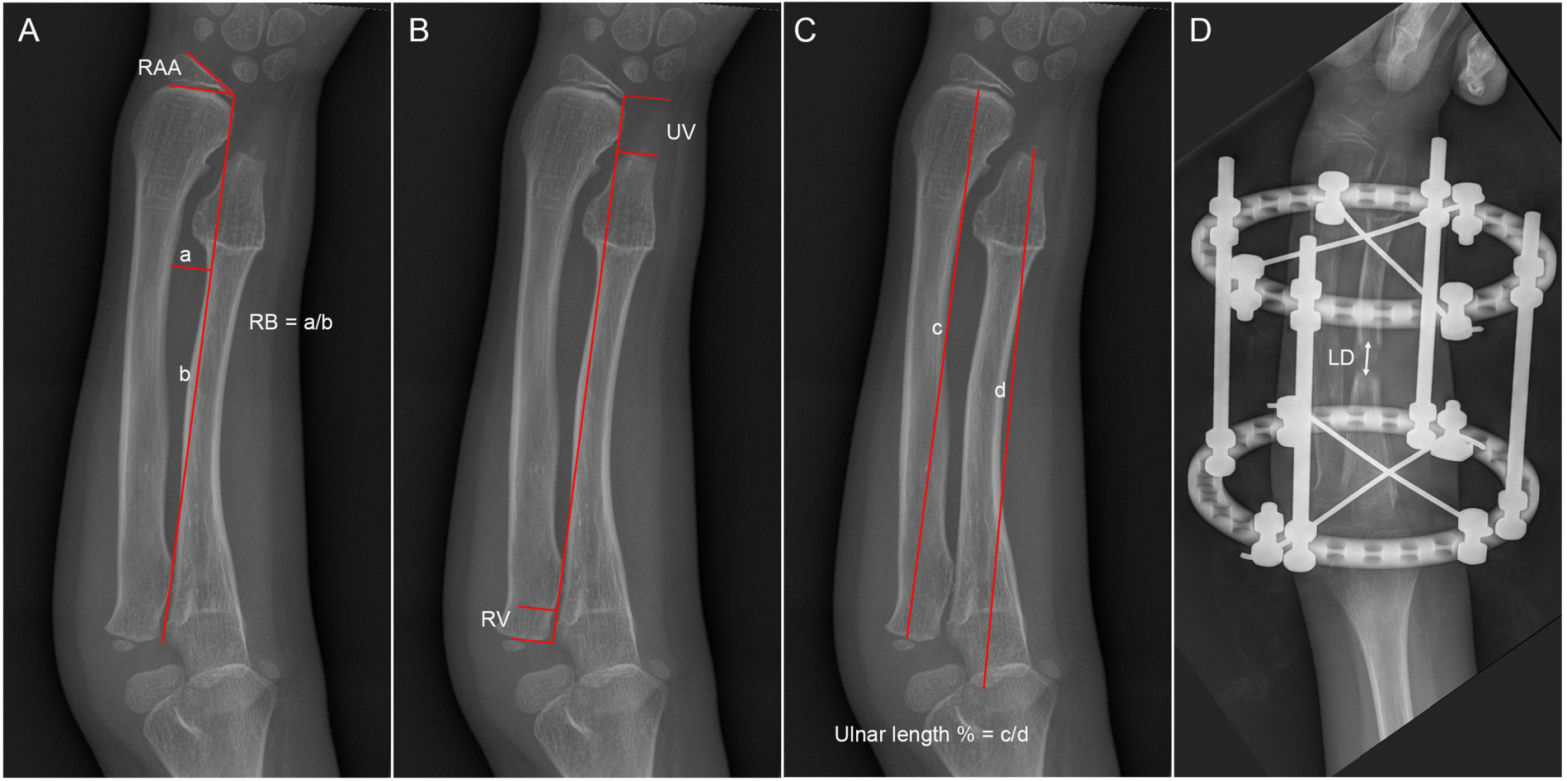
Diagrams showing the radiological measurements. **(A)** The radial articular angle (RAA) and the radial bow (RB); **(B)** the ulna variance (UV) and the radial variance; **(C)** the ulnar length percent; and **(D)** the lengthening distance (LD).

Forearm deformity was assessed on anteroposterior and lateral radiographs described by Burgess and Cates (10): radial articular angle (RAA), ulnar length percent, ulna variance (UV), radial variance (RV), and radial bow (RB) (Figure 1). The RAA is defined as the angle formed between the distal articular surface of the radius and the longitudinal axis of the forearm. The ulnar length percent was calculated by dividing ulnar length (styloid process tip to olecranon edge) by radial length (proximal-to-distal physeal centers). UV is defined as the perpendicular distance between the distal end of the ulna and the distal radial growth plate. A positive UV indicates that the ulna extends beyond the radius distally. RV represents the perpendicular distance from the radial head to the coronoid process tip. A positive RV signifies that the proximal radius projects farther than the coronoid process. The lengthening distance (LD) is the distance between the proximal and distal fragments of the ulna on the first follow-up radiograph after the final extension (Figure 1). Pre- and postoperative elbow/forearm range of motion were recorded.

We used GraphPad Prism 10.5.0 in the descriptive and statistical analyses. Continuous variables are presented as mean ± standard deviation. Normality of distribution was assessed using the Shapiro–Wilk test. For normally distributed data, paired-sample *t*-tests were employed to compare functional and radiographic parameters between preoperative and final follow-up measurements. Non-normally distributed variables were analyzed using Wilcoxon signed-rank tests. *p* < 0.05 was considered statistically significant.

## Results

The study included 12 pediatric patients, 6 girls and 6 boys, with a total of 12 forearms affected. The average age was 7.6 ± 2.9 years (range, 3.2–13). The average follow-up time was 63.8 ± 15 months (range, 26–94). Two patients were Masada type I, six were Masada type IIb, two were Masada type IIa, one was type IVa, and one was type IVb. The mean LD was 2.5 ± 0.7 cm (range, 1.4–4.0), while the duration of distraction was 141 ± 62.5 days (range, 57–265). The average distraction index was 6.0 ± 2.1 days/mm (range, 2.9–8.7). The patients' demographic information is shown in Table 2.

**Table 2:** The patients’ demographic information.

Case	Gender	Age at surgery (years)	Side	Masada classification	Length gain (cm)	Duration of distraction (days)	Distraction index (days/mm)	Follow-up time (months)
1	F	9	R	I	2.0	87	4.4	70
2	F	4.8	L	II b	2.3	102	7.8	94
3	M	5.3	R	II a	2.6	114	4.4	61
4	F	8.6	R	II b	3.2	265	8.3	64
5	F	6	R	IIb	2.5	140	5.6	62
6	M	13	R	I	3	87	2.9	26 (to maturity)
7	M	7.2	L	IVa	1.4	57	4.1	70
8	F	5.3	L	IIb	1.5	130	8.7	66
9	M	7.7	L	IIb	4.0	137	3.4	64
10	F	11	R	IVb	2.1	138	6.6	68
11	M	3.2	R	IIa	2.8	231	8.3	60
12	M	9	L	IVb	2.7	206	7.6	61

At the last follow-up, the pre-RAA was 29° ± 5.9°, while the post-RAA was 30° ± 6.3°. There was no significant difference between them. The ulnar length percent, UV, RV, and RB showed statistically significant improvements. The pre-ulnar length percent was 0.91 ± 0.05, while the post-ulnar length percent was 1.0 ± 0.08 (*p* < 0.001). The pre-UV was −1.1 ± 0.5, while the post-UV was −0.4 ± 0.5 (*p* < 0.001). The pre-RB was 8.8 ± 1.2, while the post-RB was 7.6 ± 1.4 (*p* < 0.001). The pre-RV was 0.7 ± 0.6, while the post-RV was 0.4 ± 0.8 (*p* = 0.02). The detailed radiographical information is shown in Table 3.

**Table 3:** Radiologic results of patients.

Case	Before surgery						The last follow-up					
	RAA	Ulna length %	UV (cm)	RB (%)	RV (cm)	Radial head	RAA	Ulna length %	UV (cm)	RB (%)	RV (cm)	Radial head
1	20	0.95	−1.2	8.0	0.5	G	23	1.1	0	6.8	0.4	G
2	33	0.8	−0.5	9.8	2.3	P	44	0.87	−0.7	10.3	3.0	P
3	32	0.9	−0.8	10.4	0.7	P	27	0.98	−0.1	9.0	0.4	P
4	26	0.93	−0.7	8.1	0.4	F	25	1.08	0	8.0	0.2	G
5	28	0.95	−0.9	7.7	0.5	F	28	1.05	−0.1	5.6	0.3	G
6	30	0.94	−1.1	7.8	0.1	G	28	1.08	−0.2	6.1	0	G
7	31	0.95	−1.3	9.3	0.5	F	34	1.06	−0.2	7.3	0	G
8	23	0.9	−1.6	8.5	0.3	P	27	0.99	−1.1	7.7	0	G
9	39	0.95	−1.6	8.2	0.5	F	33	1.03	−0.8	7.6	0	G
10	20	0.98	−0.4	6.9	0.8	P	23	1.1	0	6.1	0.1	G
11	30	0.86	−1.8	9.8	0.4	F	37	0.91	−1.6	7.5	0.2	G
12	36	0.86	−1.4	10.5	1.3	P	35	1.1	−0.2	9.7	−0.2	G

The patients demonstrated markedly restricted forearm rotation and impaired elbow flexion to varying extents prior to surgery. Postoperative assessment revealed improved forearm supination and elbow pronation mobility to varying extents after the ulnar lengthening procedure. However, there was no statistically significant improvement in the other ranges of motion. The pre-supination was 56° ± 10.0°, while the post-supination was 67° ± 9.4° (*p* < 0.001). The pre-pronation was 54° ± 9.3°, while the post-pronation was 65° ± 7.8° (*p* = 0.003). The pre-flexion was 117° ± 9.8°, while the post-flexion was 121° ± 9.3° (*p* = 0.06) (Table 4).

**Table 4:** Clinical assessment of the patients.

Case	Supination–pronation (°)		Flexion–extension of the elbow (°)		Complications
	Preoperative	At the last follow-up	Preoperative	At the last follow-up	
1	75–60	85–75	125-full	125-full	None
2	50–50	45–45	100-full	95-full	None
3	45–50	60–60	110-full	115-full	None
4	65–60	70–65	120-full	130-full	Infection
5	55–55	70–65	125-full	125-full	None
6	55–60	65–65	130-full	130-full	None
7	55–45	65–60	125-full	125-full	None
8	60–60	75–70	115-full	125-full	None
9	60–65	70–70	120-full	120-full	None
10	60–55	65–70	110-full	120-full	None
11	55–55	70–65	120-full	120-full	None
12	35–30	65–60	100-full	120-full	None

**Figure 2:**
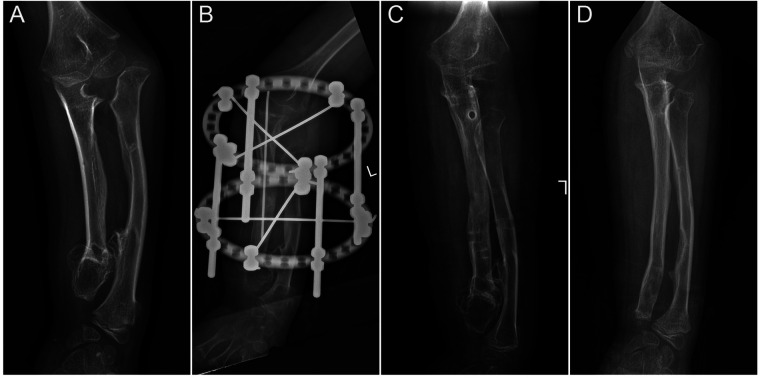
The preoperative and postoperative radiographical results of case 12. **(A)** The preoperative radiograph; **(B)** postoperative day 1 radiograph; **(C)** 1-month post-external fixator removement radiograph; and **(D)** 5-year postoperative radiograph.

**Figure 3:**
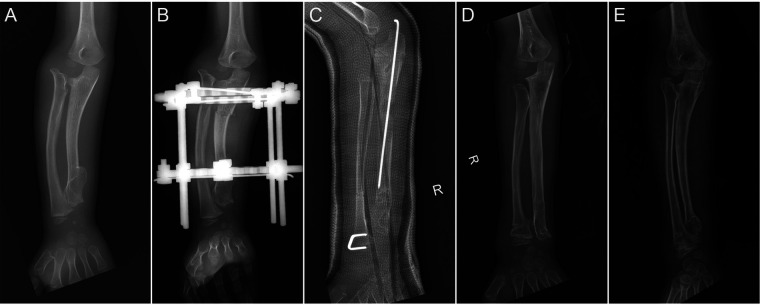
The preoperative and postoperative radiographical results of case 3. **(A)** The preoperative radiograph; **(B)** postoperative day 1 radiograph; **(C)** 1-month post-external fixator removement radiograph; **(D)** 3-year postoperative radiograph; and **(E)** 5-year postoperative radiograph.

Concentric reduction of the radial head was achieved in all patients with Masada type I deformities and in 9 out of 10 patients with radial head subluxation or dislocation following the ulnar lengthening procedure (Figure 2). During long-term follow-up, one case exhibited recurrent dislocation at the humeroradial joint (Figure 3). A pin tract infection occurred in one pediatric patient but resolved following pin care and oral antibiotic administration. Non-union or neurovascular complications were not observed in any of the patients.

## Discussion

Forearm deformities are the most common cause of dysfunction in patients with MHE. The presence of osteochondromas in the ulna and radius disrupts normal bone growth, leading to progressively worsening forearm deformities and functional impairments (11). This condition poses significant therapeutic challenges, particularly in patients who develop radial head dislocation. In this study, we found that ulnar lengthening significantly improves deformities and partially restores forearm pronation and supination in patients with severe ulnar shortening, radial bowing, and progressive radial head dislocation. However, while early surgical intervention can successfully reduce radial head dislocation, the procedure shows limited efficacy in cases of severe ulnar shortening with established dislocation.

Currently, there are various surgical approaches for treating forearm deformities in MHE patients. Depending on the number, location, and size of osteochondromas, procedures such as excision of ulnar/radial exostoses, radial corrective osteotomy, or distal radial hemiepiphysiodesis can be performed to correct deformities, improve forearm function, and relieve pain (5, 12, 13). Since MHE-related forearm deformities result from the combined effects of multiple osteochondromas, surgeons often need to adopt combined surgical strategies to provide individualized treatment for patients (14). In our study, concurrent resection of distal ulnar or radial osteochondromas was performed in all nine cases during the ulnar lengthening procedures.

Ulnar lengthening by distraction osteogenesis has been a prevalent treatment option in the management of forearm deformities associated with MHE (15, 16). The primary goal was to lengthen the shortened ulna, reduce or prevent progressive radial head dislocation, and improve both forearm alignment and joint function (17). Hsu et al. (18) found that ulnar lengthening alone is an acceptable procedure in children younger than 10 years, considering the remodeling potential and functional improvement. An analysis of 12 cases with a 32-month mean follow-up revealed that early ulnar lengthening is the key to achieving spontaneous reduction of the radial head in all patients without the need for corrective osteotomy or tumor excision (19). In our study, all five cases with radial head subluxation achieved successful reduction following ulnar lengthening, with no instances of recurrence. In contrast, only two of five patients (40%) with complete dislocation attained reduction postoperatively, although similarly without recurrent displacement. This evidence suggests that timely surgical management represents the key preventive strategy against progression to frank dislocation in children with radial head instability.

While numerous studies have reported successful reduction of radial head dislocation following ulnar lengthening procedures, the follow-up periods were generally short. In our study, case 2 presented with severe ulnar shortening (ulnar length percent = 0.8) and concomitant radial head dislocation during the initial visit to our institution. Despite undergoing ulnar lengthening procedures that achieved an ulnar length percent exceeding 1.1, spontaneous reduction of the radial head was not attained. This suggests that in cases with prolonged radial head dislocation, the radiocapitellar joint loses its normal anatomical configuration. Even if reduction is achieved, maintaining stable reduction becomes challenging, with a high propensity for recurrent dislocation. In addition, since abnormal growth patterns of the ulna and radius remain uncorrected, long-term dislocation appears inevitable. Given that dislocation of the humeroradial joint is a gradual process, short-term follow-up results can be highly misleading. Unlike most studies with a follow-up period of two to five years, our follow-up extends beyond five years or until skeletal maturity (8, 18, 20). This provides more compelling evidence regarding the efficacy of ulnar lengthening in treating or preventing radial head dislocation.

Furthermore, some researchers have investigated the osteotomy site selection and optimal lengthening extent in ulnar lengthening procedures. Lu et al. (8) proposed that the interosseous membrane, which connects the distal two-thirds of the ulna and radius, along with the proximal oblique cord, plays a crucial stabilizing role during pronation–supination movements. Their findings support the proximal third of the ulna osteotomy, as these critical structures remain intact. Huang et al. (9) found that proportional ulnar length recovering to the normal value of 1.1 would be used as a scale to decide the amount of ulnar lengthening for radial head dislocation in pediatric patients with hereditary multiple exostoses. In our study, all osteotomies were performed at the proximal third of the ulna with a proportional ulna lengthening of >1.0. However, in three pediatric cases, a progressive decrease in ulnar length percent was observed during forearm development, suggesting that aggressive ulnar lengthening may warrant consideration for younger patients with significant residual growth potential.

This study has several inherent limitations that must be acknowledged. Firstly, the retrospective design with a limited sample size restricts the strength of our conclusions. Secondly, the heterogeneous cohort composition—encompassing varying Masada classifications—introduces confounding variables that may impact outcome interpretation. Future prospective studies with larger, more homogeneous cohorts are warranted to validate the clinical efficacy of ulnar lengthening procedures.

## Conclusion

Early ulnar lengthening surgery remains a reliable treatment option for patients with MHE who develop severe forearm shortening secondary to ulnar involvement.

## Data Availability

The raw data supporting the conclusions of this article will be made available by the authors, without undue reservation.
